# Longitudinal assessment of health-related quality of life after SARS-CoV-2 infection and the associations with clinical and social characteristics in a general practice population

**DOI:** 10.1186/s12955-024-02301-7

**Published:** 2024-10-09

**Authors:** Rinske van den Hoek, Karin Hek, Isabelle Bos, Eelko Hak, Liset van Dijk

**Affiliations:** 1 Nivel, Netherlands Institute for Health Services Research, P.O. Box 1568, Utrecht, 3500 BN The Netherlands; 2 University of Groningen, Groningen, The Netherlands

**Keywords:** COVID-19, Health-related quality of life, post-COVID condition, Patient reported outcome measures, Electronic health records

## Abstract

**Background:**

We aimed to investigate the longitudinal impact of COVID-19 and the effects of clinical and psychosocial factors, accounting for post-COVID conditions (PCC), on the mental and physical aspect of health-related quality of life (HRQoL) of patients diagnosed with COVID-19.

**Methods:**

Data from the Nivel Corona Cohort were used, which includes individuals with an established SARS-CoV-2 infection that received four questionnaires over a year’s time with questions regarding HRQoL (SF-12), symptoms and social characteristics. PCC was determined based on questionnaire data. Data on medical history and healthcare utilization were obtained from electronic health records from general practice. A repeated measures linear mixed model was used to explore associations between clinical and social characteristics, and the course of mental and physical HRQoL after a SARS-CoV-2 infection, taking PCC into account.

**Results:**

One hundred fifty-eight individuals of whom it was possible to determine whether they had PCC or not were included in this study. Seventy-six (48.1%) developed PCC, which was associated with a persistent reduction in both physical and mental HRQoL. Hospitalization during the acute phase of the infection had a negative impact on the physical HRQoL, which decreased over time. Females, people older than 53, and those with increased resilience and mental HRQoL before infection were more likely to report a more positive mental HRQoL over time.

**Conclusion:**

The negative association PCC has with both mental and physical HRQoL for at least six months, calls for more research to support patients with PCC.

## Background

The COVID-19 pandemic has severely impacted populations worldwide. By September 2022, over 540 million people were infected by the coronavirus (SARS-CoV-2) worldwide, with over 8 million in the Netherlands alone [1]. The virus is known to manifest differently in symptoms and severity. While some coronavirus infected patients experience no symptoms, others get seriously ill resulting in needing medical care or death [2]. The virus impacts people both physically and mentally. Social stigmatization, the isolation after being infected or potential biological mechanisms might cause mental distress [3, 4].

As time passes, more knowledge has become available on the long-term effects of COVID-19. For instance, we now know that symptoms can persist long after an infection [5]. According to the World Health Organization (WHO), it is post-COVID condition (PCC) when SARS-CoV-2 infected patients have persistent symptoms at least three months after the onset of the initial infection, and symptoms have been present for at least two months and cannot be explained by an alternative diagnosis [5]. This definition leaves some room for interpretation resulting in varying estimates between 13 and 80% on how many people have PCC, depending on the definition used [6–9]. The severity of both acute COVID-19 and persistent symptoms has shown to negatively affect health-related quality of life (HRQoL) [10–13], which is a commonly used measure for the overall physical and mental wellbeing of an individual. A decreased HRQoL has been associated with numerous negative health outcomes, job-related issues and a less active social life. Inversely, a higher HRQoL has been associated to longer, healthy lives and increased work ability, both resulting in lower societal costs [14, 15].

Besides the presence of symptoms, other factors might also impact the HRQoL after a SARS-CoV-2 infection. Factors such as resilience, social support and the ability to cope have been shown to positively affect HRQoL, as they are thought to play a role in individual differences in physical and psychological responses to distress [16–18]. In addition, studies have found inadequate health literacy, a high BMI and a low education level to be associated with a lower HRQoL [9, 19, 20]. Similarly for the presence of chronic illnesses such as diabetes or COPD, which have also been associated to a decreased HRQoL [21, 22]. However, the effect of patient characteristics on HRQoL has not yet been studied over time and in relation to a previous SARS-CoV-2 infection.

We aim to study the longitudinal impact of COVID-19 and the effects of clinical and psychosocial factors, while accounting for PCC, on the course of HRQoL in patients who have had COVID-19 [12, 13]. Insight in clinical and psychosocial factors that affect the course of HRQoL is crucial for patients and health care providers as it potentially enables the opportunity to intervene in time preventing a decrease in HRQoL. More knowledge might also increase the understanding for patients who experience long lasting effects of a SARS-CoV-2 infection.

## Method

### Setting

Nivel, the Netherlands Institute for Health Services Research, has a Primary Care Database (Nivel-PCD) in which electronic health records (EHR) of general practices are collected. Approximately 500 general practices participate in Nivel-PCD, covering around 1.8 million patients. Nivel-PCD is a longitudinal database, with an ongoing data collection and is representative of the Dutch population on patients age and sex [23]. In 2016 the Benefit, Risk and Impact of Medication Monitor (BRIMM) research infrastructure was developed [24]. This infrastructure allowed Nivel-PCD to do research by combining data from EHRs and patient reported outcomes (PROs) via surveys. Based on the principles of this infrastructure Nivel initiated a longitudinal cohort study of COVID-19 patients in 2020: the Nivel Corona Cohort [25]. More details about the recruitment and data collection can be found elsewhere [25].

### Cohort recruitment

Twenty-five general practitioners (GPs) who weekly deliver data to the Nivel-PCD were solicited to participate in cohort recruitment. They reacted positively to an earlier call in the Nivel-PCD newsletter for participants that was placed to see whether there was sufficient interest among GPs to participate in the study. Eighteen of these 25 GPs partook in this study. The participating GPs were located throughout the Netherlands in both rural and urban areas [25]. Nivel developed an algorithm to flag adult patients with a COVID-19 registration (International Classification of Primary Care [ICPC]-code R83.03) in their EHR [25]. COVID-19 could have been diagnosed by the GP, but the registration also entailed results from testing facilities from the Municipal Health Services (GGD) if the patient consented to this information being shared with the GP. A trusted third party (TTP), which held the encryption key for the pseudonymized patients, sent the flagged decrypted patient-IDs to the associated GP. The GP checked the patient’s eligibility for participation. Patients were found eligible when they had COVID-19, remained in the same practice and were able to fill in the questionnaire, thus there would be no language barriers, personal problems or too severe disease burden. The GP returned the flagged and checked patients to the TTP, who invited the patient to participate in this study on behalf of their GP. Patient recruitment occurred between January and September 2021.

### Data collection

Over a year’s time, recruited patients received four questionnaires containing questions regarding the SARS-CoV-2 infection. The first questionnaire was sent right after the agreement to participate (Q1, baseline), and then subsequently after three months (Q2), after six months (Q3) and after one year (Q4). Therefore, Q1 does not refer to the moment of the SARS-CoV-2 infection, but to the beginning of participation. The questionnaires contained questions on morbidity, treatment, clinical outcomes and lifestyle, as well as information on the impact of COVID-19 in terms of QoL and ability to work. The first questionnaire also contained questions regarding the participants’ situation prior to their SARS-CoV-2 infection. For this study, we used data from Q1, Q2 and Q3. We did not use Q4 as this data was not available during the time of analyses. Data from these questionnaires were collected between January 2021 and April 2022 depending on when the patient was recruited.

The pseudonymized data obtained from the questionnaires was, when a patient gave informed consent for this, linked to the patient’s GP EHRs collected in the Nivel-PCD. From the EHRs we extracted information on (co-)morbidities (ICPC-1 coded) that were registered in 2019, thus prior to the infection.

For this study we included patients who had a recent SARS-CoV-2 infection less than three months prior to receiving the baseline questionnaire (Q1). Patients who had more than three months between infection and the first questionnaire were excluded from this specific study to prevent recall bias on quality of life before the infection.

### Outcome measure: health-related quality of life

The main outcome was health-related QoL (HRQoL) before the infection and at the time of Q1, Q2 and Q3. HRQoL was measured using the mental component summary and the physical component summary scores of the Short Form (SF)-12 [26]. Summary scores were calculated ranging from 0 to 100, with lower scores meaning worse quality of physical or mental health [26–28]. The average score was considered 50 and a deviation of 10 points below or above the average was considered as the standard deviation.

### Post-COVID condition

The PCC definition was based on a Delphi consensus reported by the World Health Organization [29]. An individual was classified as having PCC when they reported at least one symptom three months after the initial infection and reported not to be recovered after three months. Individuals were classified as non-PCC when they reported to be recovered within three months. If there was missing data or inconsistencies in the answer (not recovered, but also no report of symptoms), it was not possible to determine whether these individuals had PCC and they were thus excluded from the analyses.

### Clinical and sociodemographic covariates

We selected clinical and sociodemographic characteristics that could be related to the HRQoL based on literature [Appendix 1, Table 4: Covariate description and classification].

All covariates were measured at Q1. Number of chronic comorbidities was pre-defined in Nivel-PCD based on constructed disease episodes [30]. Level of education was considered low if individuals discontinued their education after primary school, within the first three years of secondary school or if they completed preparatory secondary vocational education (VMBO). The middle category consisted of individuals who completed senior general secondary education (HAVO) or university preparatory education (VWO) or vocational education (MBO). A high level of education included individuals who completed university or university of applied sciences. Social support in case of problems was calculated on the subset “Support in case of problems” of the SSL-12-I, a Dutch validated scale to measure social support [31, 32]. Resilience was calculated on the nine-item Resilience Evaluation Scale (RES), which was validated in the Dutch population [31]. If one or more questions on the scales were not answered, we substituted the missing value by the mean of the non-missing answers. If none of the questions were answered, the final score was left empty and considered missing. Health literacy was calculated using three brief screening questions [33]. If the mean score of these screening questions was below 2, we considered the individual to have inadequate health literacy. Migration background was defined as at least one of the participants’ parents being born outside of the Netherlands. In case of missing data, we performed row wise deletion prior to the analyses.

### Data analysis

First, we described variables, using number and percentage for categorical variables and mean values and standard deviations for continuous variables. With Excel, we visualized separately the mental and physical HRQoL at timepoints prior to the infection, in Q1, Q2 and Q3 for individuals with and without PCC. For each time period in the questionnaires, the difference between people with and without PCC was assessed for both the physical and mental HRQoL using chi-square analyses.

To assess the association between the selected covariates and the course of HRQoL after the infection, we used a linear mixed-effects model for repeated measures analysis. We performed a backward elimination procedure with a p-value of 0.2 for a first selection of variables that were potentially associated with the outcome [34]. Single regression analyses were additionally performed to verify whether any previously deleted, potentially relevant characteristics were incorrectly eliminated from the model based on statistical significance at a p-value of 5% or lower. Statistical significance was assumed at α = 5%. As the effect of covariates on the HRQoL might differ directly after the infection and months after the infection, an interaction with time was analyzed for each covariate after variable selection and added to the model when this interaction was statistically significant. All analyses, except the visualization, were performed using Stata/SE 16.1.

## Results

### Descriptive statistics

**Fig. 1:**
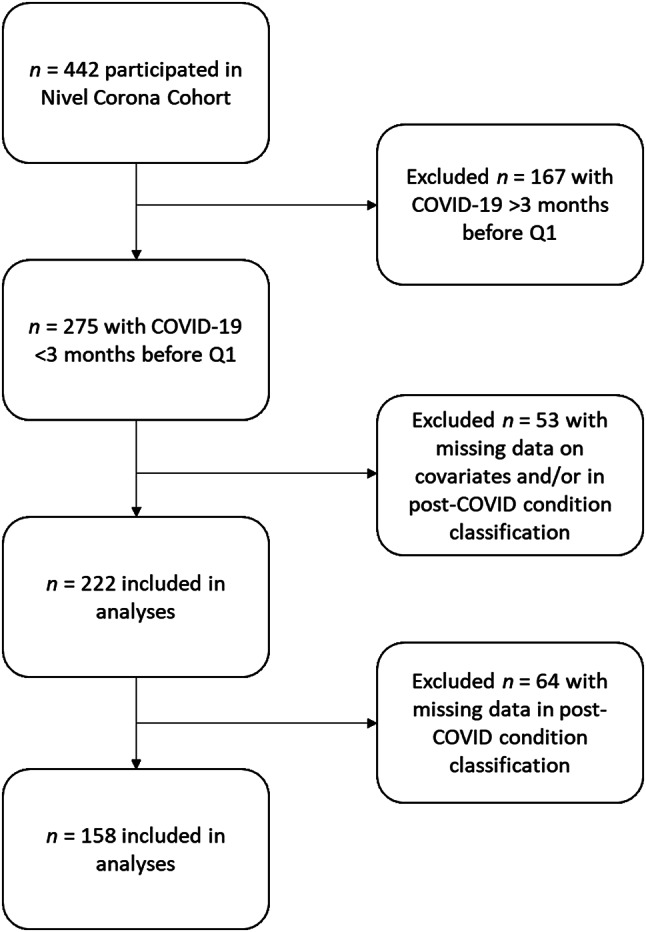
Flowchart with details on sample selection

We included 158 individuals who had been infected with SARS-CoV-2 less than three months before Q1 (Fig. 1) and for whom enough data was acquired to assess whether they had PCC or not. Compared to the total population in the Nivel Corona Cohort, our sample was slightly older (54.1 years old in this sample versus 51.5 years old in the Nivel Corona Cohort) and included relatively more females (63.3% in this sample and 60.9% in the Nivel Corona Cohort). Little less than half of these individuals (*n* = 76, 48.1%) developed PCC and *n* = 82 (51.9%) did not develop PCC.

Table 1 shows the characteristics of the included patients. The majority experienced hardly any symptoms (19.0%) or symptoms comparable to a cold (44.3%) during the acute phase of the SARS-CoV-2 infection.

**Table 1:** Patient characteristics

	Total	PCC	No PCC
Total, *n*	158	76	82
Female sex, *n* (%)	100 (63.3%)	45 (59.2%)	55 (67.1%)
Age (years), mean [*SD*]	54.1 [12.3]	55.0 [12.6]	53.3 [12.0]
Post-COVID condition			
Post-COVID condition	76 (48.1%)		
No post-COVID condition	82 (51.9%)		
Self-perceived severity acute phase, *n* (%)			
Hardly any to no symptoms	30 (19.0%)	7 (9.2%)	23 (28.1%)
Comparable to cold	70 (44.3%)	27 (35.5%)	43 (52.4%)
Very ill, but not hospitalized	46 (29.1%)	30 (39.5%)	16 (19.5%)
Hospitalized	12 (7.6%)	12 (15.8%)	0 (0%)
Chronic illnesses, *n* (%)			
0	90 (57.0%)	42 (55.3%)	48 (58.5%)
1	27 (17.1%)	10 (13.2%)	17 (20.7%)
2+	41 (26.0%)	24 (31.6%)	17 (20.7%)
Body Mass Index, *n* (%)			
< 18.5 (underweight)	0 (0.0%)	0 (0%)	0 (0%)
18.5–25 (healthy weight)	54 (34.2%)	16 (21.1%)	38 (46.3%)
25–30 (overweight)	83 (52.5%	46 (60.5%)	37 (45.1%)
> 30 (obese)	21 (13.3%)	14 (18.4%)	7 (8.5%)
Level of education, *n* (%)			
Low	39 (24.7%)	30 (39.5%)	9 (11.0%)
Middle	52 (32.9%)	23 (30.3%)	29 (35.3%)
High	67 (42.4%)	23 (30.3%)	44 (53.7%)
Social support, mean [*SD*]	8.6 [3.3]	8.8 [3.3]	8.4 [3.4]
Resilience, mean [*SD*]	27.1 [5.5]	26.0 [6.2]	28.1 [4.6]
Inadequate health literacy, *n* (%)	2 (1.3%)	0 (0%)	2 (2.4%)
Migration background, *n* (%)	11 (7.0%)	7 (9.2%)	4 (4.9%)
Smoking, *n* (%)			
No, never smoked	76 (48.1%)	35 (46.1%)	41 (50.0%)
No, but former smoker	72 (45.6%)	35 (46.1%)	37 (45.1%)
Yes, current smoker	10 (6.3%)	6 (7.9%)	4 (4.9%)

### The course of HRQoL after COVID-19

**Fig. 2:**
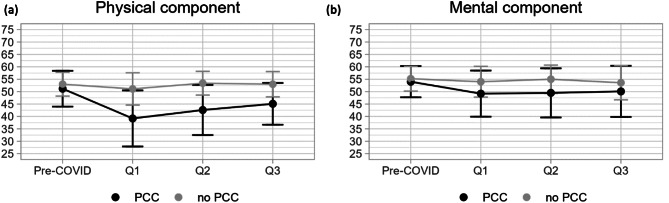
(**A**) The course of the physical component of health-related quality of life before the coronavirus infection, at Q1, Q2 and Q3. (**B**) The course of the mental component of health-related quality of life before the coronavirus infection, at Q1, Q2 and Q3

Figure 2 shows the physical (Fig. 2A and Appendix 2: Table 5) and mental (Fig. 2B and Appendix 2: Table 6) HRQoL over time in individuals with and without PCC. Before the infection (pre-COVID), the physical component of the HRQoL (Fig. 2A) was not statistically different between individuals with and without PCC [Appendix 2: Table 7]. At all timepoints after the infection, the physical HRQoL was significantly lower for individuals with PCC compared to those without, although the physical HRQoL did increase over time for patients with PCC. At the baseline questionnaire (Q1), after the infection, the physical HRQoL for patients with PCC was below 40, indicating a difference compared to the mean score of the Dutch normative sample which is approximately 50 [28].

For individuals without PCC, the physical HRQoL restored to the level from before the infection in Q2.

The standard deviation of the physical HRQoL was largest at Q1 for both the PCC and the non-PCC group 11.3 and 7.5 respectively [Appendix 5: Table 5] and decreased over time.

Similarly, the mental component (Fig. 2B) did not differ significantly between individuals with and without PCC before the infection, but did so at all timepoints after the infection [Appendix 2: Table 7]. The mean mental HRQoL was at the lowest level at baseline for patients with PCC and remained statistically lower at Q2 and Q3 compared to the same patients before the infection.

For individuals without PCC, no statistical difference was measured between mental HRQoL pre infection and Q1, Q2 and Q3 respectively. The standard deviation for the mental component of HRQoL increased for individuals with PCC from pre-COVID (6.3) to Q3 (10.3). For those without PCC, the largest deviation was also measured at Q3, being 6.2.

### Factors associated with HRQoL: outcomes of a mixed-effects repeated measures model

For the physical aspect of HRQoL (Table 2), higher physical HRQoL before the infection was associated with an increased HRQoL at Q1 (0.66, *p* < 0.01), but this positive effect neutralized three months later. Similar to Fig. 2A, individuals with PCC had a significantly lower HRQoL (-7.02, *p* < 0.01) after the infection which did not change significantly over time. Patients who were very ill or hospitalized during the acute SARS-CoV-2 infection had a significantly lower physical HRQoL at Q1 (-5.90, *p* < 0.01 and − 9.66, *p* < 0.01 respectively). No significant decrease nor change in HRQoL over time was seen for individuals whose SARS-CoV-2 infection was comparable to a cold. For individuals who were hospitalized we observed a partial recovery in HRQoL at Q2 (5.93, *p* = 0.02) and Q3 (6.83, *p* = 0.01) compared to Q1. Resilience had a small positive effect (0.23, *p* = 0.01) on the physical HRQoL in general, this effect did not change over time. In the single regressions [Appendix 3: Table 8] a BMI higher than 25 was associated to a lower physical HRQoL. This effect was not statistically significant in the multiple regression model.

**Table 2:** Multiple regression of demographic and clinical characteristics with the course of the physical component of health-related quality of life after a SARS-CoV-2 infection

Variable	Multiple regression	
	Coef. (95% CI)	*p* value
Sex		
Male	ref	ref
Female	-0.90 (-2.94; 1.13)	0.38
Age		
18–53	ref	ref
54 and over	0.39 (-1.61; 2.40)	0.70
Time		
Q1	ref	ref
Q2	25.94 (15.65; 36.24)	< 0.01*
Q3	27.96 (15.95; 39.97)	< 0.01*
Post-COVID condition		
No post-COVID condition	ref	ref
Post-COVID condition	-7.02 (-9.11; -4.92)	< 0.01*
Physical HRQoL prior to SARS-CoV-2 infection	0.66 (0.46; 0.87)	< 0.01*
* Interaction time Q2*	-0.45 (-0.64; -0.26)	< 0.01*
* Interaction time Q3*	-0.50 (-0.72; -0.28)	< 0.01*
Self-perceived severity acute phase		
Hardly any to no symptoms	ref	ref
Comparable to cold	-0.77 (-3.97; 2.42)	0.63
* Interaction time Q2*	-1.01 (-4.09; 2.07)	0.52
* Interaction time Q3*	0.01 (-3.53; 3.55)	1.00
Very ill, but not hospitalized	-5.90 (-9.46; -2.35)	< 0.01*
* Interaction time Q2*	1.72 (-1.59; 5.04)	0.31
* Interaction time Q3*	4.38 (0.64; 8.11)	0.02*
Hospitalized	-9.66 (-14.93; -4.40)	< 0.01*
* Interaction time Q2*	5.93 (1.09; 10.77)	0.02*
* Interaction time Q3*	6.83 (1.47; 12.19)	0.01*
Resilience	0.23 (0.05; 0.40)	0.01*
Body Mass Index		
18.5–25 (healthy weight)	ref	ref
25–30 (overweight)	-1.89 (-4.02–0.24)	0.08
> 30 (obese)	-3.09 (-6.23–0.05)	0.05

CI = confidence interval, HRQoL = health-related quality of life

*Statistically significant

Considering mental HRQoL (Table 3), it was found that resilience (0.18, *p* = 0.02), older age (3.35, *p* < 0.01), female sex (1.75, *p* = 0.03) and the mental HRQoL prior to the infection (0.70, *p* < 0.01) were associated to a more positive mental HRQoL. Having PCC (-3.78, *p* < 0.01), having a migration background (-3.78, *p* = 0.01) and having one comorbidity (-2.28, *p* = 0.03) were negatively associated with the mental HRQoL right after the coronavirus infection. None of these covariates interacted with time.

**Table 3:** Multiple regression of demographic and clinical characteristics with the course of the mental component of health-related quality of life after a SARS-CoV-2 infection

Variable		Multiple regression		
		Coef. (95% CI)	*p* value	
Sex				
Male		ref	ref	
Female		1.75 (0.15; 3.34)	0.03*	
Age				
18–53		ref	ref	
54 and over		3.35 (1.74; 4.96)	< 0.01*	
Time				
Q1		ref	ref	
Q2		0.71 (-0.56; 1.99)	0.28	
Q3		0.42 (-1.04: 1.87)	0.58	
Post-COVID condition				
No post-COVID condition		ref	ref	
Post-COVID condition		-3.78 (-5.29; -2.26)	< 0.01*	
Mental HRQoL prior to SARS-CoV-2 infection		0.70 (0.57; 0.83)	< 0.01*	
Resilience		0.18 (0.030; 0.32)	0.02*	
Migration				
No		ref	ref	
Yes		-3.78 (-6.72; -0.85)	0.01*	
Chronic comorbidities				
None		ref	ref	
One		-2.28 (-4.31; -0.25)	0.03*	
Two or more		-1.64 (-3.54; 0.25)	0.09	

CI = confidence interval, HRQoL = health-related quality of life

*Statistically significant

## Discussion

The twofold aim of this study was to assess the longitudinal impact of a SARS-CoV-2 infection on the mental and physical HRQoL and to identify clinical and psychosocial factors associated with the HRQoL after a SARS-CoV-2 infection.

Having PCC was the major factor in explaining a decreased HRQoL after a SARS-CoV-2 infection. Both the physical and mental aspect of HRQoL were lower for patients with PCC compared to individuals without PCC after the infection. This effect lasted at least six months after the infection. Besides the general lower HRQoL in the PCC group, our findings also show a larger standard deviation after the infection, which indicates heterogeneity in how people are affected by PCC.

Other studies reported similar findings. An Irish study by O’Kelly et al., which was similar in the number of included individuals and HRQoL measuring tool, showed a lower physical component of HRQoL, but not a lower mental component of HRQoL a year after the SARS-CoV-2 infection for patients with PCC compared to those without [35]. On the other hand, Seeßle et al. showed a small reduction in the mental component [36]. Compared to the physical component, the difference in the mental component was smaller, yet present in our results as well. This sentiment was also seen in systematic reviews where, despite differences in measuring tools, times of assessment, different definitions of PCC or the use of a specific population (e.g. only hospitalized patients), a decreased HRQoL was seen in patients with PCC [10, 11].

Multiple other factors affected the HRQoL as well. We observed that a higher HRQoL before the infection was associated to an increased physical HRQoL at baseline. This strengthens the hypothesis that better general wellbeing makes a positive difference on how people will experience an acute illness, such as COVID-19 [37]. The severity of the acute COVID-19, especially in hospital admitted individuals, was also associated to a lower physical HRQoL compared to those who had barely any to or no symptoms. Being admitted to the hospital during the acute phase has been shown to negatively affect the HRQoL elsewhere as well [38, 39].

Resilience was found to have a small positive effect on both the physical and mental component of HRQoL. Our findings contribute to a large body of evidence showing that resilience has a positive effect on QoL in general, during the COVID pandemic [18] and also in combination with several other illnesses [17, 40] and in patients with PCC [41].

Our results showed a positive association between age and mental HRQoL, where older age was associated with an increased mental HRQoL compared to people under the age of 54. Young adults were more vulnerable to a decreasing mental health in the pandemic compared to older individuals, as has been shown in several studies [42–44]. Contrarily, we found no effect for age on the physical component, while studies do report decreasing physical wellbeing in elderly [11, 45]. A potential explanation might be that our study corrects for the physical constraints of having PCC, which might eliminate negative physical aspects of aging.

Having a migration background was associated to a decreased mental HRQoL compared to people without a migration background. This finding was also seen in the (non-)COVID population in other studies [46, 47] and can be explained by experiencing difficulty in facing an illness in a foreign country and a lack of having traditional support networks.

Chronic illnesses were expected to be related to a lower HRQoL as physical health is an important contributing factor to a decreased HRQoL [21, 22]. Our data showed that having one chronic illness was in fact associated to a decreased physical HRQoL, but this effect was not seen in individuals with multiple chronic comorbidities. It is difficult to assess the impact of the chronic illness. For example, eczema is considered a chronic illness, but to what extent people are limited in their daily life differs between patients [48]. The variety in severity and impact of chronic illnesses might therefore explain our findings.

### Strengths and limitations

Our study has several strengths. Via our infrastructure where we recruited patients via the GP, we were able to combine patient reported outcomes and GP EHR-data. We were able to include information on a wide range of factors that are associated with the HRQoL from both the patient and the clinical perspective. Moreover, our methods provided the opportunity for longitudinal analysis, and we were able to study two distinguished aspects of HRQoL at different time points.

We sampled the data from a population-based cohort, and we therefore included a wide variety of individuals with differences in severity of SARS-CoV-2 infection. On the other hand, due to the nature of questionnaires it is more difficult to include illiterate individuals as is reflected in the low number of people with inadequate health literacy or with a migration background. As described in previous publications, participants in the Nivel Corona Cohort are slightly older and more often female than the group that was initially invited to participate in the cohort [49]. Moreover, we only included individuals in the analyzes of whom we could determine whether they had PCC or not. Although our sample is therefore not representative for the Dutch population as a whole, these factors seemed not to substantially influence the outcome measure.

Another possible limitation of our data was that it was unknown whether we were dealing with reinfections, first infections or later infections at baseline nor whether the patient experienced a new SARS-CoV-2 infection during follow-up. A new infection that occurred over the course of this study may have decreased the HRQoL over time. In this study vaccination data was not sufficiently available, thus we were not able to take into account whether participants were (fully) vaccinated or not.

Potential recall bias may have been caused by the fact that we retrospectively asked about the HRQoL prior to the infection. Patients with PCC may memorize their quality of life different than non-PCC patients which may have led to an overestimation of the effect of having PCC on the HRQoL. By only selecting patients with a recent SARS-CoV-2 infection that was no longer than three months before completing the first questionnaire, we limited this bias as much as possible, although it still might be present. Another limitation might be that we were not able to take possible lockdown effects into account as we did not have a control group from a time period without lockdowns.

### Clinical implications

This study showed the large and long-lasting impact of having PCC on the patients’ wellbeing. Therefore, our findings call for more research on interventions and treatments for PCC to improve the physical and mental HRQoL. Given that people with PCC can have a broad spectrum of symptoms, it is difficult to identify interventions appropriate for everyone. However, a potential opportunity might be found in improving resilience. Our findings did show that resilience had a slight positive impact on the HRQoL in this cohort. Implementing interventions that enhance resilience, such as training programs, might aid patients with PCC [50], yet other studies would have to show whether such a training would still be useful after the infection.

## Conclusion

In conclusion, our study shows that having PCC has a profound negative impact on both the mental and the physical aspect of the HRQoL, which lasts for at least six months after the infection. A small, but positive effect was found in resilience, female sex and in people older than 53 on the mental HRQoL. Our findings call for more research on the PCC patient group to improve interventions as these patients seem to experience negative long-term effects from the SARS-CoV-2 infection.

## Appendix 1: Covariate description and classification

**Table 4:** Covariate description and classification

Variable	Unit
Age	0: 18–53 1: 54 and older
Sex	0: male 1: female
HRQoL prior to infection, mental or physical	Score scale: 0-100
Post-COVID condition (PCC)	0: no PCC 1: PCC 2: unknown
Self-perceived severity of illness during the acute phase of the SARS-CoV-2 infection	1: hardly to no complaints 2: complaints comparable to a cold 3: very ill, but not hospitalized 4: hospitalized
Number of chronic diseases described in the GP EHRs prior to the infection (30)	0 1 2+
Level of education	1: low education level 2: middle education level 3: high education level
Body mass index (BMI)	< 18.5: underweight 18.5–25: healthy weight 25–30: overweight > 30: obese
Experienced social support in case of problems (SSL-12-I, (31))	Score scale: 4–16
Resilience score (Resilience Evaluation Scale, (32))	Score scale: 0–36
Health literacy (Chew et al. screening questions (33))	0: inadequate health literacy 1: adequate health literacy
Migration background	0: no 1: yes

## Appendix 2: Quality of life in individuals with and without post-COVID condition

**Table 5:** Mean physical quality of life measured in three questionnaires for individuals with and without post-COVID condition

Physical	Post-COVID condition		No Post-COVID condition	
	Mean	SD	Mean	SD
Pre-COVID	51.2	7.2	53.0	4.8
Q1	39.2	11.3	51.1	6.5
Q2	42.6	10.1	53.4	4.8
Q3	45.1	8.4	53.0	5.1

SD = standard deviation

**Table 6:** Mean mental quality of life measured in three questionnaires for individuals with and without post-COVID conditions

Mental	Post-COVID condition		NO Post-COVID condition	
	Mean	SD	Mean	SD
Pre-COVID	54.0	6.3	55.2	5.0
Q1	49.2	9.3	54.0	6.2
Q2	49.5	9.9	55.0	5.7
Q3	50.1	10.3	53.6	6.9

SD = standard deviation

**Table 7:** Chi-square analyses between individuals with and without post-COVID condition

Period	*p* value
Pre-physical component	0.054
Physical component Q1	< 0.01
physical component Q2	< 0.01
physical component Q3	< 0.01
Pre-mental component	0.17
Mental component Q1	< 0.01
Mental component Q2	< 0.01
Mental component Q3	0.030

## Appendix 3: Single regression analyses

**Table 8:** Single regression analyses of all covariates on the physical component and mental component of quality of life

			Physical component		Mental component	
			**Coef. (95% CI)**	***p*** **value**	**Coef. (95% CI)**	***p*** **value**
Sex (male = reference)						
	Female		-0.29 (-2.88; 2.30)	0.83	0.40 (-1.86; 2.66)	0.73
Age (18–53 = reference)						
	54 and over		-1.83 (-4.31; 0.66)	0.15	1.96 (-0.19; 4.13)	0.07
Time (Q1 = reference)						
	Q2		2.82 (1.58; 4.07)	< 0.01	0.71 (-0.56; 1.99)	0.28
	Q3		3.82 (2.40; 5.24)	< 0.01	0.39 (-1.07; 1.86)	0.60
Physical HRQoL prior to SARS-CoV-2 infection			0.46 (0.26; 0.66)	< 0.01		
Mental HRQoL prior to SARS-CoV-2 infection					0.77 (0.62; 0.92)	< 0.01
Symptoms (Hardly any to no symptoms = reference)						
	Comparable to cold		-2.45 (-5.60; 0.71)	0.12	0.91 (-2.05; 3.88)	0.55
	Very ill, but not hospitalized		-6.21 (-9.58; -2.85)	< 0.01	-1.25 (-4.43; 1.93)	0.44
	Hospitalized		-11.42 (-16.27; -6.58)	< 0.01	-0.38 (-5.00; 4.24)	0.87
Social support			-0.29 (-0.67; 0.08)	0.12	-0.31 (-0.64; 0.012)	0.06
Resilience			0.37 (0.15; 0.59)	< 0.01	0.40 (0.21; 0.59)	< 0.01
PCC			-9.68 (-11.72; -7.65)	< 0.01	-4.82 (-6.87; -2.77)	< 0.01
Migration background (No = reference)						
	Yes		-0.90 (-5.75; 3.95)	0.72	-2.82 (-7.074; 1.43)	0.19
Chronic comorbidities (No = reference)						
	1		0.35 (-3.01; 3.72)	0.84	-2.30 (-5.27; 0.68)	0.13
	2 or more		-3.20 (-6.10; -0.30)	0.03	-1.48 (-4.03; 1.078)	0.26
Education (low = reference)						
	Middle		3.78 (0.57; 6.98)	0.02	0.75 (-2.14; 3.64)	0.61
	High		5.54 (2.50; 8.58)	< 0.01	1.76 (-0.98; 4.50)	0.21
BMI (18.5–25 (healthy weight) = reference)						
	25–30 (overweight)		-4.57 (-7.15; -1.98)	< 0.01	0.49 (-1.91; 2.88)	0.69
	> 30 (obese)		-7.75 (-11.52; -3.97)	< 0.01	0.027 (-3.49; 3.55)	0.99
Smoking status (never = reference)						
	Former smoker		-1.98 (-4.53–0.56)	0.13	-0.46 (-2.71–1.79)	0.69
	Current smoker		-4.25 (-9.68–1.19)	0.13	1.83 (-2.81–6.47)	0.44

CI = confidence interval, HRQoL = health-related quality of life

## Data Availability

The datasets used and/or analyzed during the current study are available under conditions.
